# The effects of the Norwegian Coordination Reform on the use of rehabilitation services: panel data analyses of service use, 2010 to 2013

**DOI:** 10.1186/s12913-016-1564-6

**Published:** 2016-08-05

**Authors:** Lars C. Monkerud, Trond Tjerbo

**Affiliations:** 1 Norwegian Institute for Urban and Regional Research, Centre for Welfare and Labour Research, Oslo and Akershus University College of Applied Sciences, P.O. Box 4, St. Olavs plass, Oslo, 0130 Norway; 2 Departement of Health Management and Health Economics, Institute of Health and Society, Faculty of Medicine, University of Oslo, P.O. Box 1089, Blindern, Oslo, 0317 Norway

**Keywords:** Integrated care, Health care reform, Rehabilitation services, Primary care reform, Specialist and primary health care coordination, Municipal co-financing

## Abstract

**Background:**

In 2012 the Norwegian Coordination Reform was implemented. The main motivation was to encourage municipalities to expand local, primary health care services. From 2012 to 2014, under the Municipal Co-Financing regime, municipalities were obliged to cover 20 % of the costs of health services provided at the specialist (hospital) level. Importantly, use of rehabilitation services in private institutions was not part of the cost-sharing mechanism of Municipal Co-Financing. Rehabilitation services may be seen as quite similar in nature whether they be provided by municipalities, hospitals or private institutions. Thus, with rehabilitation patients readily “transferrable” between levels, the question is whether the reform brought with it a sought after shift towards *more* municipal rehabilitation and *less* specialist rehabilitation.

**Methods:**

Data from the Norwegian Patient Register and from Statistics Norway/KOSTRA were utilized to gauge annual expenditures and inputs in specialist, municipal and private institution rehabilitation services respectively. Fixed effects and first difference regression analyses for the period 2010–2013 were carried out to account for certain time-invariant traits of municipalities and/or hospital regions, and results were adjusted for contemporaneous trends in local needs.

**Results:**

Expenditures in specialist rehabilitation services declined sharply (typically by 8–10 %) from 2011 (pre-reform) to 2012 (post-reform), while expenditures in private rehabilitation services rose markedly in the same period (typically by 42–44 %). The results do not suggest any general expansion of municipal rehabilitation services.

**Conclusions:**

The results of the analyses suggest that municipalities shift *away* from the use of specialist rehabilitation services and *towards* the use of rehabilitation services in private institutions since the latter becomes relatively cheaper (free-of charge) than both municipal and specialist services in post-reform periods (as specialist services come at a cost to municipalities post-reform). While the main goal of the reform has not materialized the results nevertheless suggest that incentives (of cost-shifting) do play a significant role in rehabilitation service use.

**Electronic supplementary material:**

The online version of this article (doi:10.1186/s12913-016-1564-6) contains supplementary material, which is available to authorized users.

## Background

### Norwegian Coordination Reform and rehabilitation services

Implemented in 2012, the main goal of the Norwegian Coordination Reform (CR) [1]^1^ was to reduce overall health care costs and facilitate the coordination of the primary and specialist levels of the Norwegian health care system. Coordination difficulties often follow financial and regulatory divisions between specialist and primary health care [2]. This is also the case in Norway, where the provision of specialist and primary health care services is at different organizational levels and financed through different budgets, a condition particularly pronounced in rehabilitation services. The subsequent division of responsibilities between the specialist and primary health care level within rehabilitation has been described as unclear [3], in the sense that both levels maintain much the same rehabilitation capabilities and competencies. Moreover, as both municipalities—charged with primary health care responsibilities—and specialist health care hospital regions are reasonably well suited to provide such services, the incentives to shift costs onto the other part perhaps constitutes the core “coordination problem” in this area.

In this way, the case of rehabilitation services constitutes something of a litmus test for the CR in terms of the prospects for simple coordination. For instance, a finding that the reform has *not* had the expected effect on these services, i.e. a decline in specialist rehabilitation and an increase in primary rehabilitation, would highlight a serious shortcoming of the reform. This is not necessarily because the reform was tailored specifically for rehabilitation services, but rather because this is where the potential for simple coordination should be particularly evident.

An important feature of the CR was Municipal Co-Financing (MCF). Under this provision, lasting from 2012 to 2014, municipalities were obligated to cover 20 % of the costs associated with a given set of medical Diagnostic-Related Groups (DRGs), including hospital rehabilitation services. The overall motivation for introducing co-financing was that it would provide municipalities with an incentive to expand (primary) health care provided at the municipal level, in the hope that this in turn would lessen the need for more expensive specialist health care, and thereby reduce overall health costs and improve health outcomes [3, 4]. In addition, because it adheres to the Lowest Effective Level (LEL) of care principle, and because municipalities may naturally integrate local medical services with other municipal social and care services, any growth at this level would be particularly beneficial [3, 4].

In this article, we analyze the effects of MCF on the use of both specialist and primary level rehabilitation services, along with its effects on the procurement of rehabilitation services in *private institutions*. Importantly, there has always been in Norway a relatively small private sector offering rehabilitation services. Equally important, the use of private rehabilitation services was, somewhat peculiarly, exempt from the MCF scheme (i.e. the continuation of financing through hospital revenues). The role of private rehabilitation institutions has thus been somewhat politically controversial, and strong incentives for the municipalities to expand their own services could undermine the need for these private sector institutions. In any case, we would expect that at least initially private rehabilitation services would expand under the MCF regime owing to municipal preferences for “free-of-charge” services, insofar as private services may really substitute for services provided by hospitals and/or by municipalities themselves. In the ensuing analyses, we employ regression analysis using annual municipal-level aggregated data from the Norwegian Patient Registry (NPR) [5] (providing data on specialist level and private rehabilitation expenditures) and the Municipality-State-Reporting information system (KOSTRA) [6] (providing data on local health service resources and local demographics) over the period 2010–2013.

The article is organized as follows. To start, we briefly place our research questions within the broader research field dealing with the relationship between the primary and specialist level health care sectors, and describe the overall Norwegian institutional setting, with a particular emphasis on the specifics of the CR and MCF regime. Subsequently, we develop hypotheses concerning the expected *demand* for rehabilitation services at different levels, both pre- and postreform, as this is responsive to both changes in (i) (demographic) needs, and (ii) to (pre- and postreform) service prices. The following sections then present our data and empirical strategy, and the results from the statistical analyses. In the regression analysis, we trace the changes in demand for different rehabilitation services, employing both fixed effects and first-difference specifications in order to account for local heterogeneity over the period of study. Although we cannot rule out that changes in the use of different rehabilitation services are in fact the result of other confounding factors, we do believe that the introduction of the MCF regime is the most important contemporaneous driving force, and our analyses gives us some leeway in evaluating the impact of confounding trends due to other factors. We discuss our findings and their implications before providing concluding remarks.

### The relationship between health care levels, the Norwegian institutional setting, and the CR: implications for rehabilitation services

Often described as a decentralized National Health Service (NHS)-type system, the fundamental features of the Norwegian health care system resemble those of the UK NHS, although Norwegian municipalities play a relatively more important role than do their UK counterparts. For example, the responsibility for Long Term Care (LTC) services and community health services lies at the municipal level. Provision of general practitioner (GP) services is also a municipal responsibility, although GPs are usually not municipal employees as such. Rather, they are independent service providers operating in a contractual relationship with the municipalities [7].^2^ For their part, the revenues of the municipalities consist of block grants from the central state, taxes on personal income and wealth, and other specific grants, such as grants to municipalities in rural areas. There is the redistribution of tax income among municipalities to reduce inequalities, and the most important part of the block grant is a per capita component adjusted for centrality and inequalities in the demand for services. Thus, the design of the system serves to adjust for municipal needs and factors affecting the ability of municipalities to deliver services.

For the most part, the financing of somatic specialist health care is through a combination of an adjusted per capita-based block grant and Activity-Based Financing (ABF) based on the DRGs. Even though the Regional Health Authorities (RHAs) are free to choose the model they deem best for financing hospitals in their catchment area, all have opted for the same model as the central state uses to fund the RHAs themselves. Consequently, while the ABF share of the total funding of specialist health care has varied between 40 and 60 % over the last 15 years, in the shorter more recent period under study (2010–2013), it has remained at 40 % throughout.

Introduced in 2012, the CR’s main elements were: (i) MCF, whereby the municipalities had to cover 20 % of the running hospital costs for medical DRGs; (ii) a discharge arrangement where municipalities were charged NOK 4000 per day from when a patient was classified as ready for discharge by the hospital; and (iii) new municipal acute bed units (MAUs). The MAUs are intermediate units introduced to reduce use acute admissions to hospitals. By law, both RHAs and municipalities must have a unit responsible for the coordination of rehabilitation services. Municipalities are also required to devise individual plans for each patient/user in need of long-term care and to provide coordinated health services [8]. One of the primary goals of these regulations concerning rehabilitation was to strengthen coordination within both the primary and specialist levels, as well as between the two levels. The integration of health and social care is at the municipal level, and this, in combination with the proximity of local authorities to users, means that municipalities are well suited to provide high-quality “low-tech” rehabilitation services. Moreover, in the white paper first introducing the CR, the then Minister of Health stressed in the foreword the importance of enabling the municipalities to provide rehabilitation services [4]. In other words, there are clear arguments in favor of an increase in the use of municipal rehabilitation, both as far as quality and costs are concerned.

Furthermore, we have argued that rehabilitation services rendered at different levels (specialist, municipal, and private) are “substitutable” in a very simple fashion. Numerous studies show that primary health care interventions, seen as *preventive* interventions, may have positive effects in terms of better long-term health outcomes and lower overall health care costs [9]. Nevertheless, such studies, and associated recommendations, will typically point to gains from the substitution of simpler primary care interventions for costly and complex treatment at later stages, i.e. gains in terms of better health outcomes and/or lower overall health costs only secured after some time [9–16].

In contrast, when analyzing the use of rehabilitation services at different levels, we look at the substitution of quite *similar* services across levels, which may or may not have effects in terms of better outcomes in the longer run. In other words, the focus of the analysis is on services that are *easily shifted between levels* and *should* be *strengthened at the municipal level* (given municipal services have certain qualities), but that *might not* shift in this fashion given the peculiarities of regulation, financing regime, and reform design. This facilitates the analysis of potential incentive effects, such as those stemming from the MCF, and ensures that other more “medical”-related coordination problems (e.g. the correct ratio of heart surgery to exercise) are less of a concern. In sum, both the apparent political significance of the rehabilitation area and certain simplifying aspects of the area itself serve to motivate the importance of the analysis.

Before the CR, use of specialist rehabilitation services was ostensibly “free of charge” for municipalities. With the reform from 2012 onward, and following the introduction of MCF, municipalities were required to cover 20 % of all specialist-level health care costs. The main motivation for this was to provide municipalities with an incentive to expand their own services, including rehabilitation services. Lastly, as noted earlier, nonprofit private rehabilitation institutions play an important role in the institutional configuration of rehabilitation services in Norway. Importantly, the MCF scheme did not cover the use of private rehabilitation, with its financing continuing through hospital block grants.

We now hypothesize about the *incentive effects* in the pre- and postreform periods. These, of course, do not necessarily reflect the *aspirations of the reform*. The first issue concerns the likely patterns of service provision demand as we *change* from the pre- to the postreform period. As the use of private rehabilitation providers is “free of charge” for municipalities, with costs covered through hospital block grants—and given *both* the use of (postreform) specialist rehabilitation and that the expansion of own capabilities comes at a cost—we expect *municipal demands for private rehabilitation services to increase at the expense of their demands for specialist and municipal rehabilitation*. In addition, we would expect that the *demand for municipal provision would not increase much in absolute terms* regardless. Even if there are certain intrinsic qualities regarding municipal services compared with specialist and private services, the former are likely considered “too expensive” vis-à-vis readily available alternatives, i.e. specialist services at what is likely a fraction of the cost (80 % of the specialist service price at any rate), and free private services.^3^

Before we turn to a description of the data and the empirical analyses, we briefly consider that the hypotheses developed above focus on municipal incentives. While such a focus is in line with the “official” working mechanism of the reform, we also believe this is appropriate for two reasons. First, municipal incentives change dramatically and clearly as the reform sets in and as described. In contrast, hospitals are only partly able to maximize disposable revenues and/or minimize outlays. However, if hospitals prefer long-term “major activity”, they will certainly favor a situation where municipalities purchase specialist services, insofar as specialist DRG prices are accurate. Conversely, they will most disfavor the situation where municipalities demand private rehabilitation, as this will also deplete current disposable funds (that nearly match activity-based refunds—recall that private rehabilitation is not part of the ABF system). Given the limited incentives of hospitals, with their largely refund-based financing system, in the following analyses and interpretations, we focus on municipal incentives. Moreover, it should be clear from the discussion that a finding in accordance with the main hypotheses outlined above (dealing with municipal incentives) is in fact a *conservative* one (given that hospital incentives should drive patterns in the opposite direction). In addition, the analyses also offer controls for certain invariant characteristics of hospitals as well as municipalities and private service providers (e.g. rigid capacity limits).

Municipalities can act on their incentives in three ways. First, although the relationship between the municipality and the GP is contractual (the vast majority of GPs are contractors), the municipality likely has some influence over the GP’s decisions. Second, they can prioritize sending patients that come from the hospital to a private rehabilitation hospital, rather than providing municipal services or using public hospital services. Third, they can refer patients who have received care in the new MAUs who are in need of further rehabilitation to private institutions and public hospitals or municipal rehabilitation.

A final point is that in analyzing the changes in the patterns of rehabilitation service provision, we should naturally adjust for possibly confounding changes in local health care needs during the period of study (proxied by local demographics in our study). Thus, a natural, albeit auxiliary, set of hypotheses is that changes in service levels should be less sensitive to needs when services are costly. In other words, we expect that *needs variables are more important predictors of specialist rehabilitation use before the reform than after the reform*. This is also an effect of the relative prices of the different alternative rehabilitation services available to the municipalities.

## Methods

### Data

In the empirical analyses, we use aggregate data from the Norwegian Patient Registry (NPR) recording (i) annual municipality-level utilization of six specialist rehabilitation DRGs and (ii) annual municipality-level use of private rehabilitation services (24 h stays) over the period 2010–2013.^4^ We calculate

(i) the *annual costs of specialist rehabilitation DRGs per capita* in each municipality by annually aggregating individual DRG diagnoses multiplied by the annual DRG weight attached to each DRG type multiplied by the annual DRG price^5^ [17] and dividing by the annual average municipal population;
(ii) the *annual costs of private rehabilitation services per capita* in each municipality by annually aggregating individual stays in private rehabilitation institutions multiplied by the average price per stay (NOK 2886 in 2012) and dividing by the annual average municipal population.^6^

As a measure of the level of municipal rehabilitation services, we use Statistics Norway/KOSTRA records on annual man-hours for physio- and ergotherapists in municipal employment, and calculate

(iii) the *annual municipal sum of man-hours worked by physio- and ergotherapists per capita* by dividing total man-hours by the annual average municipal population [18].^7^

To compare the level of service costs over time and the cost sensitivities to local needs and resources, we deflate the measure of DRG use using the Norwegian Consumer Price Index [19]. Thus, the costs presented in the tables and analyses are in 2010 NOK (or its log).

In addition, we use data from Statistics Norway for the period 2010–2013 to gauge local levels of rehabilitation needs [20]. Specifically, we use the annual municipal population shares of 67–79 year-olds and those 80 years old and over as measures of local needs. This is because these population groups will reasonably require the most extensive health care, including rehabilitation services. We also employ a measure of reported local crime rates (per 10,000 inhabitants), and the rate of deaths per capita as proxies for local living conditions. This is because lower living conditions will reasonably entail heavier use of health services, including rehabilitation services. Additionally, we extract from the KOSTRA data the annual local population to calculate the per capita rates of the service-level variables (i)–(iii) above. Table 1 provides descriptive statistics for the 409 municipalities with valid values for these variables.^8^

**Table 1:** Descriptive statistics. Service use and municipal needs levels. By year (2010–2013). *N* = 409.^a^

Variable	Year	Mean	St. dev.	Min.	Max.
Specialist level (hospital) rehabilitation services (2010 NOK per inabitant)^b^	2010	251.84	123.81	34.78	1137.12
	2011	239.71	109.56	40.17	1057.75
	2012	212.38	96.01	25.40	891.81
	2013	200.13	92.11	21.13	1122.35
Rehabilitation services in private institutions (2010 NOK per inhabitant)^c^	2010	18.08	8.48	2.26	80.19
	2011	17.61	10.94	1.67	189.63
	2012	27.73	27.01	3.20	187.26
	2013	29.25	23.49	2.18	179.46
Municipal rehabilitation services (physiotherapist and ergotherapist man-years per 10,000 inhabitants)	2010	3.74	1.87	0.05	32.00
	2011	3.93	1.79	0.04	16.08
	2012	4.10	1.78	0.07	23.51
	2013	4.30	1.89	0.07	18.51
Share of municipal population aged over 80	2010	0.05	0.01	0.02	0.10
	2011	0.04	0.01	0.02	0.09
	2012	0.04	0.01	0.02	0.09
	2013	0.04	0.01	0.02	0.09
Share of municipal population aged 67–79	2010	0.08	0.02	0.05	0.15
	2011	0.08	0.02	0.05	0.16
	2012	0.09	0.02	0.05	0.17
	2013	0.09	0.02	0.05	0.18
Deaths per 1000 inahbitants	2010	8.51	2.17	3.62	22.02
	2011	8.38	2.11	3.03	21.12
	2012	8.38	2.20	3.53	20.42
	2013	8.14	2.17	3.90	22.73
Reported crimes per 10,000 inahbitants	2010	79.50	36.52	11.40	210.20
	2011	76.68	36.03	10.80	208.90
	2012	75.29	36.07	9.40	205.40
	2013	74.77	35.85	10.70	234.80

*Source:* Norwegian Patient Registry and Statistics Norway

^a^ Results weighted by municipal population

^b^ Comprises DRG-462A: Complex rehabilitation, DRG-462B: Ordinary rehabilitation, DRG-462C: Other rehabilitation, DRG-462O: Unspecified rehabilitation, DRG-932O: Policinic rehabilitation and DRG-998O: Group based patient recovery

^c^ annually aggregated individual stays in privat rehabilitation institutions times average price-per-stay (NOK 2886 in 2012)

The most apparent pattern in Table 1 is the sharp decline in the use of specialist rehabilitation services from an average of NOK 252 per inhabitant in 2010 (prereform) to NOK 200 in 2013 (a 20 % decline). In addition, the use of rehabilitation services in private institutions increases dramatically over the same period, from NOK 18 per inhabitant in 2010 to NOK 29 in 2013, i.e. the average increases by a staggering 61 %.

Conversely, man-hour inputs in municipal rehabilitation services increase only from 3.73 per 10,000 inhabitants in 2010 to 4.30 per 10,000 inhabitants in 2013 (i.e. an increase of 15 %). The empirical question is whether these results hold when we adjust for contemporaneous trends in local needs and look specifically at the patterns *within* the typical municipality (by way of fixed-effects and first-difference model specifications).

### Model specification

As we wish to trace the changes in the use of different rehabilitation services, we estimate variants of the following panel (fixed effects) model:

1 $$ \log {\left({y}_{it}\right)}^k={\alpha}_0^k+{\displaystyle \sum_{T=2011}^{2013}{\alpha}_T^k}\cdot Yea{r}_{Tt}+{\displaystyle \sum_{m=2}^{409}{\beta}_m^k}\cdot Mu{n}_{mi}+{\displaystyle \sum_{c=1}^4{\gamma}_c^k\cdot \log \left( Need{s}_{cit}\right)}+{\varepsilon}_{it}^k $$

That is, we estimate a set of *k* = 3 equations where *y* is a particular measure of rehabilitation service use (*k* = specialist, private, municipal) in municipality *i* in year *t* (*t* = 2010, 2011, 2012, 2013); and *needs* is a vector of *c* = 4 municipal needs variables (*c* = two measures of shares of elderly, death rates and crime rates), with *γ* a vector of associated parameters. The inclusion of indicators for *N* = 409 municipalities, i.e. the *Mun* indicator variables, means that we effectively control for any traits of municipalities—or the relationship between the individual municipality and the health region—that are stable over the relevant period (such as stable capacity constraints, say). In addition, we estimate the equations in terms of logs of the variables, so that we may interpret effects as elasticities, and so as to get at a more reasonable and normal scale for measured changes in outcomes in particular: As can be seen in Table 1, outcome levels (i.e. rehabilitation service use) are (naturally) heavily skewed to the right, while this is also so, although less pronounced, for the *needs* variables.^9^ The tendency for distributions to be right-skewed also applies to relative changes in variables (not shown), and a log-transformation (of change rates) seems to remedy such problems considerably: As can be seen in Additional file 1: Table S1, minimum and maximum values of log-transformed changes seem to be distanced quite symmetrically around their mean values.

Much of the “distributional concerns” discussed above also have to do with the fact that municipalities are predominantly small in Norway^10^ and population rates of use of rehabilitation services are in themselves relatively small. This causes the variables (the dependent variables in particular) to be poorly measured as “substantive changes” in quite many of the observations. Specifically, variable values in most cases, i.e. in the smaller localities, will reflect large changes due to quite random movements of very few individuals (in and out of age groups, in and out of municipal employment). Thus, when reporting descriptive statistics and when estimating the equations, we weight observations (*it*, municipality-years) by the average annual municipal population, and interpretations are in terms of effects for the “average sized municipality” (around 12,000 inhabitants in 2012).

Our primary interest lies in the estimates of the *α*_*T*_ coefficients, i.e. the effects associated with the *Year*_*T*_indicator variables (*T* = 2011, 2012, 2013, with 2010 the reference category). Although we are not able to take account of all other possible factors that coincide with the reform, other than changes in municipal needs and time-invariant municipal characteristics, we can think of no other systematic factor that should drive service use in a particular direction during the period under study. Accordingly, we interpret the *α*_*T*_ as “reform effects”.

## Results

We present results from the estimation of variants of equation (1) in Table 2. First, in models without covariates, i.e. models (A), we estimate significantly different service levels in reform years (2012 and 2013) as compared to the reference year (2010). For example, as compared to levels in 2010, average use of specialist services decreases by 14 % by 2012 and by 20 % by 2013.^11^ Conversely, the use of rehabilitation services in private institutions increases sharply in the same manner, while there is less pronounced increase in the use of municipal services. Joint tests of the differences between 2011 and 2012 levels, which may be interpreted as the *adjustment* going into the reform, reveal that effects in all service sectors are substantial and highly significant.^12^ On the other hand, levels do not change substantively or significantly in the pre-reform period from 2010 to 2011, save for municipal rehabilitation services.^13^ Moreover, joint tests of differences between levels in 2012 and 2013, i.e. *within* the reform, are smaller than changes from 2011 to 2012, suggesting that there is little adjustment *both before and after* the reform, but some adjustment going into the reform.^14^

**Table 2:** Use of rehabilitation services. OLS (models A and B) and IV (model C) regressions with municipality fixed effects. *N* = 1636.^a^

	Use of specialist level (hospital) rehabilitation services (log)			Use of rehabilitation services in private institutions (log)			Use of municipal rehabilitation services (log)		
	(A)	(B)	(C)	(A)	(B)	(C)	(A)	(B)	(C)
*Year*_*2011*_ (=1)	−0.03	−0.03	−0.02	−0.07	−0.06	−0.04	0.07 ***	0.05 **	0.00
	(0.02)	(0.02)	(0.03)	(0.05)	(0.05)	(0.04)	(0.03)	(0.02)	(0.03)
*Year*_*2012*_ (=1)	−0.15 ***	−0.15 ***	−0.11 **	0.25 ***	0.27 ***	0.32 ***	0.12 ***	0.06 *	−0.05
	(0.02)	(0.03)	(0.06)	(0.05)	(0.06)	(0.09)	(0.03)	(0.03)	(0.08)
*Year*_*2013*_ (=1)	−0.22 ***	−0.21 ***	−0.15	0.34 ***	0.36 ***	0.47 ***	0.18 ***	0.07	−0.13
	(0.02)	(0.05)	(0.10)	(0.06)	(0.07)	(0.16)	(0.03)	(0.05)	(0.15)
Share of pop. 67–79 (log)		0.04	−0.96		−0.52	−2.97 *		1.40 **	3.81 **
		(0.48)	(1.11)		(0.63)	(1.74)		(0.63)	(1.71)
Share of pop.80+ (log)		0.36	0.96		−0.86	−4.60 ***		0.21	1.29
		(0.29)	(1.01)		(0.80)	(1.69)		(0.45)	(1.58)
Deaths per inhab. (log)		0.02	−0.70		−0.02	0.85		0.05	−1.55
		(0.07)	(1.04)		(0.10)	(1.44)		(0.09)	(1.39)
Reported crimes per inhab. (log)		0.04	−0.02		0.13	0.05		−0.16	−0.10
		(0.10)	(0.11)		(0.14)	(0.14)		(0.12)	(0.12)
R^2^	0.78	0.78	0.76	0.84	0.84	0.82	0.84	0.84	0.79

^a^ Results weighted by municipal population. All regressions include municipality indicators (parameter estimates not shown). Reference year is 2010 (Y_2010_ = 1). Robust standard errors in parentheses

* *p* < 0.10, ** *p* < 0.05, *** *p* < 0.01

In the next column (B) we add needs levels (*Needs*_*c*_) in the individual municipality-year, since service use should arguably be affected by changes in needs. We observe that needs—in the form of shares of elderly people—are positively, but not significantly, related to use of specialist and municipal service levels, but negatively related to use of rehabilitation services in private institutions. Likely, this reflects that specialist rehabilitation, more than rehabilitation in private institutions, is to some extent more attuned to the needs of the elderly. Regardless, estimated *α*_*T*_ largely retain their previous values, suggesting that there are pronounced “reform effects”.

Next, in models (C) we apply instrumental variables (IV) estimation, instrumenting the two elderly shares variables and the death rate variable by their lagged values from three years back. The idea is that a straightforward ordinary least squares (OLS) specification, as in model variants (A) and (B), will give biased estimates of needs effects, since there is most likely some *reverse causality* in the relationship between needs and service use. Specifically, a particular pattern of service use, induced by the CR, say, might arguably influence the survival of inhabitants and demographics as such (young vs. elderly) in the individual municipality.

Thus, observed correlations between needs and service use may not reflect a true causal effect from the former to the latter. Moreover, since the mentioned needs variables are likely endogenous in this way, not only will estimates for needs effects be biased, effect estimates for other explanatory variables that are correlated with needs—for example year effects—will also be biased. Our argument for the IV specification is that previous needs levels in part determine present needs (death rates and demographics), but not current service use, which is plausibly determined systematically by present needs only. Results for IV estimations are presented in column (C) of Table 2.^15^

From the estimations of model variant (C), as compared to (A) and (B), we observe that the positive CR effects, i.e. year effects, are somewhat more pronounced in the case of private rehabilitation services—with *α*_2012_ estimtates increasing from around 0.26 to 0.32; that the negative CR effect in specialist services is somewhat attenuated—with the estimated *α*_2012_ dropping from−0.15 to−0.11; and that the positive effect in municipal rehabilitation services shifts to a negative, albeit not significant, effect. In addition, the negative effects of shares of elderly mentioned above become more pronounced and highly significant. Despite this however, joint tests of the *α*_*T*_ estimates reveal that the pattern of pre- and post-reform *non*-adjustment and reform adjustment (from 2011 to 2012) stays much the same as in model specifications (A) and (B).^16^

**Fig. 1:**
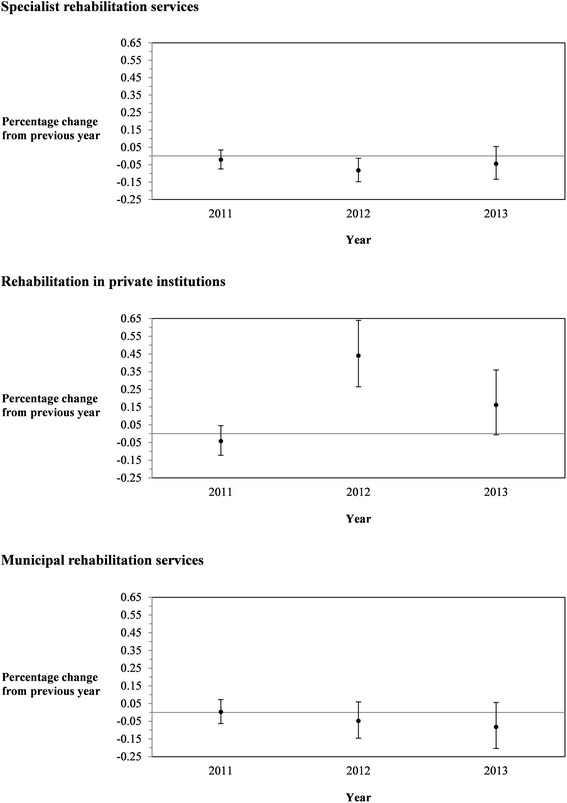
Percentage changes from previous year in the use of rehabilitation services (needs adjusted). [Legend: Based on estimates of the *α*_*T*_ in equation (1), model specification (C)]

These results are in line with the hypothesized pattern in the previous discussion: Municipalities shift use towards services that become relatively less costly. For ease of exposition, we illustrate the results from the analyses in model (C), i.e. from joint tests of *α*_*T*_ estimates, in Fig. 1. Here, annual predicted changes in the use of the different rehabilitation service types are plotted along time (year). The pattern clearly suggests that municipalities adjust their use of rehabilitation services towards those that become cheaper (i.e. towards private services going into the MCF reform in 2012) and away from those that get costlier (i.e. away from specialist services in the same period).

As a final robustness check, we estimate effects in a first-differenced specification:

2 $$ \varDelta \log {\left({y}_{it}\right)}^k={\alpha}_0^k+{\displaystyle \sum_{T=2012}^{2013}{\alpha}_T^k}\cdot Yea{r}_{Tt}+{\displaystyle \sum_{c=1}^4{\gamma}_c^k\varDelta \log {\left( Need{s}_{cit}\right)}^k}+\varDelta {\varepsilon}_{it}^k $$

The motivation for this is that error terms, *ε*_*it*_, might be serially correlated, and under such circumstances first-differencing, rather than a fixed effects specification, is a better choice [21, 22].^17^ Results are shown in Table 3. Bearing in mind that the *α*_*T*_ effects are now estimates of *changes* directly, or differences in *changes*, rather than levels, one observes in model variant (D) much the same pattern as in the fixed effects analyses in model specification (1). Specifically, there is little and insignificant change in specialist service levels from 2010 to 2011, as witnessed by the intercept estimate $\left({\widehat{\alpha}}_0=-0.03\right).$ However, there seems to be some significant decrease in the use of private rehabilitation services $\left({\widehat{\alpha}}_0=-0.07\right)$ and some increase in municipal services $\left({\widehat{\alpha}}_0=0.06\right).$

**Table 3:** Use of rehabilitation services. First-difference regressions without (model D) and with (model E) time specific needs effects (model E). *N* = 1227.^a^

	Change from previous year in use of specialist level (hospital) rehabilitation services (log)		Change from previous year in use of rehabilitation services in private institutions (log)		Change from previous year in use of municipal rehabilitation services (log)	
	(D)	(E)	(D)	(E)	(D)	(E)
Intercept	−0.03	−0.02	−0.07 ***	−0.06 **	0.06 ***	0.05
	(0.02)	(0.02)	(0.02)	(0.03)	(0.02)	(0.03)
*Year*_*2012*_ (=1)	−0.09 ***	−0.09 **	0.40 ***	0.42 ***	−0.03	0.04
	(0.03)	(0.04)	(0.06)	(0.07)	(0.03)	(0.05)
*Year*_*2013*_ (=1)	−0.03	−0.02	0.16 ***	0.13 **	−0.02	0.01
	(0.02)	(0.03)	(0.05)	(0.06)	(0.03)	(0.05)
Δ share of pop. 67–79 (log)	−0.12	0.92	−0.48	−0.43	0.76	1.05
	(0.49)	(0.89)	(0.57)	(0.90)	(0.79)	(1.83)
Δ share of pop.80+ (log)	0.53	1.34	−0.46	0.19	−0.21	−0.74
	(0.32)	(0.76)	(0.83)	(0.63)	(0.61)	(1.01)
Δ deaths per inhab. (log)	0.00	0.05	0.04	0.01	0.07	−0.08
	(0.11)	(0.11)	(0.08)	(0.11)	(0.09)	(0.12)
Δ reported crimes per inhab. (log)	−0.03	0.38 *	0.03	0.06	−0.13	−0.13
	(0.02)	(0.21)	(0.14)	(0.19)	(0.13)	(0.26)
R^2^	0.02	0.03	0.16	0.16	0.01	0.01

^**a**^ Results weighted by municipal population. Robust standard errors clustered at the municipality level in parentheses

* *p* < 0.10, ** *p* < 0.05, *** *p* < 0.01

Changes going into the reform are more pronounced: For instance, the change in the use of specialist rehabilitation services from 2011 to 2012 is estimated to be ${\widehat{\alpha}}_{2012}=-0.09$*larger* than the pre-reform change from 2010 to 2011 $\left({\widehat{\alpha}}_0=-0.03\right).$ In other words, the average 2011–2012 change in specialist rehabilitation use is estimated to be ${\widehat{\alpha}}_0+{\widehat{\alpha}}_{2012}=-0.12,$ which is comparable to what was found in the fixed effects analyses shown in Table 1. Overall, in the case of in specialist and private rehabilitation service use, the mentioned pattern of reform adjustment and pre- and post-reform *non*-adjustment also prevails in the first-differenced specification.^18^

Lastly, in model variant (E) we allow for time-specific needs effects, in order to test the hypothesis that municipal use of specialist rehabilitation services should be less sensitive to needs as services become costlier.^19^ As can be seen in column (E) in the analysis of specialist rehabilitation services, the needs effects are all positive, and significantly so for the crime rate variable.^20^ In addition, as shown in Additional file 3: Table S3, the accompanying interaction terms are by and large negative, and significantly so for the interaction between crime rate variable and the indicator variable for 2012.

This is broadly in line with the hypothesis that municipal use of (specialist) rehabilitation services should be less sensitive to needs as services become costlier (as they do in reform periods). Moreover, the pattern of reform adjustment and pre- and post-reform *non*-adjustment, evaluated by joint tests of the *α*_0_ and the *α*_*T*_ estimates, is apparent also in the time-varying needs effects specification (E). Specifically, for specialist rehabilitation services there is no significant pre-reform (2010–2011) adjustment (*p* = 0.37), a significant adjustment going into the reform and no (*p* = 0.00), and no post-reform adjustment (*p* = 0.23). The pattern is the same for use of private rehabilitation services, but with a significant *decrease* pre-reform (cf. the negative and significant, *p* = 0.02, *α*_0_), and there are no significant year-to-year changes for the use of municipal rehabilitation services.^21^

## Discussion

While there was a pronounced focus on improving and expanding rehabilitation services in the years preceding the reform, its primary goal was to provide municipalities with an incentive to expand their own services. Nonetheless, the CR does not appear to have led to a general increase in the provision of municipal rehabilitation services.

Of course, we should note some possible limitations of our study. First, our measure of municipal rehabilitation use is rather rough (see note 7). Future research should attempt to acquire better data to address this point, as the measure arguably does not encompass the full breadth of municipal rehabilitation services. Second, as we do not have information about the source of the patients admitted to private rehabilitation institutions, we cannot rule out the possibility that much of the increase stems from patients arriving from hospitals. Certainly, the length of stay for patients in hospitals decreased after the CR [23], although the waiting time for somatic patients appears to have changed less [24]. Nonetheless, a reduction in the mean LOS may also have led to a greater share of patients arriving at private rehabilitation institutions from public hospitals, but we do not know to what extent this has happened.

Another mechanism introduced in the CR was discharge arrangements where municipalities were charged a fee per day for patients, in the case that they were declared ready for discharge but could not be received by the municipalities. One way for municipalities to handle this, for example if they did not have the capacity to receive patients, was to use private rehabilitation institutions. Some of the patients provided with acute care in the new MAUs rather than in a hospital would probably also require rehabilitation services. Unfortunately, again, our data does not allow us to identify where patients come from. Other elements of the CR, in particular the new discharge arrangement, may play an important role here. Nevertheless, municipalities appear to have vastly increased their use of private providers of rehabilitation services rather than expanding their own services. This is most likely because of the relative increase in the price for specialist rehabilitation services in the wake of the CR. For example, with the MCF, the municipalities would need to cover 20 % of the costs of such services themselves. On the other hand, the use of private providers of rehabilitation services has remained free of charge throughout, which may explain the observed strong *decline* in the use of specialist rehabilitation services as the reform set in. Thus, the reform aspirations that would point toward local service expansion have not occurred. Instead, local authorities have perhaps naturally shifted their attention toward readily available and *cheaper* alternatives (private rehabilitation services).

Increased cost sharing under the MCF regime might have created stronger incentives for expanding municipal services. Nevertheless, under such an arrangement, municipalities would have had to handle *increased risks*. Demand for services will fluctuate from year to year, and for smaller municipalities, i.e. for smaller catchment areas, this will often be difficult to manage [3]. In other words, there is a trade-off between the incentives placed on agents (municipalities) and the amount of risk entailed. In the Norwegian context, where the municipalities are often small and therefore particularly susceptible to financial risk, this is a very real problem. Consequently, one way to enhance the potential for incentive schemes such as MCF—but without disproportionately increasing financial risks to an unacceptable level—would be the consolidation of local governments. However, at present the prospects for substantial local government structural reform in Norway do not look good.

Reforms that rely on incentivizing mechanisms also need to address the institutional configuration of the system. On the one hand, much of the literature [25], along with evaluations of the recent Danish structural reform [26], indicates that the potential for success from incentivizing schemes such as the MCF is low. There are few indications of a general substitution effect. Nevertheless, rehabilitation services are arguably an area where the potential could be greater. We find no effect in terms of *reform aims*, i.e. expansion of local government services, although there are indications that municipalities responded to financial incentives present in the reform. With private rehabilitation services exempt from the MCF and ABF altogether, the reform created incentives for municipalities to rely on private providers, and indeed, we observed a strong increase in the use of private rehabilitation services. On this basis, we could argue that providing municipalities with stronger incentives to expand their own services might have worked better to achieve reform goals. Counterfactually, the inclusion of private rehabilitation services in the MCF and/or larger local governments might have led to different patterns of rehabilitation service use. As argued, it seems that the institutional configuration, i.e. a strong preference for “small local government,” provides little room for implementing cost-sharing mechanisms sufficiently strong enough to have any substantial effect.

## Conclusions

Our findings suggest that municipalities shifted *away* from the use of specialist rehabilitation services and *toward* the use of rehabilitation services in private institutions because the latter are relatively cheaper (i.e. free-of charge) than either municipal or specialist services in the postreform period. In addition, there is little evidence of any substantial expansion of the municipal rehabilitation service sector following from the CR and the MCF mechanism. Since ours is a temporal field study, and neither an experiment, nor one in which a separate control group is available, we cannot rule out that other unobserved confounding factors contemporaneous to the reform are what is driving the patterns of rehabilitation service use. However, to our knowledge there are few other systematic factors correlated with the initiation of the reform, but *not* stemming form the reform themselves, that could explain the observed patterns. In addition, we find that pre- and post-reform trends are largely absent, suggesting that other confounding factors, potentially at play throughout the period of study, are not as important as the reform itself. While our findings mean that the main goal of the reform has not been met, the results nevertheless suggest that the cost-shifting incentives still play a significant role in rehabilitation service use.

## Abbreviations

ABF, Activity Based Financing; CR, Coordination Reform; DRG, Diagnostic-Related Groups; IV, Instrumental Variables; KOSTRA, Municipality-State-Reporting system (*Kommune-Stat-Rapportering*); LEL, Lowest Effective Level; LTC, Log Term Care; MCF, Municipal Co-financinig; NHS, National Health Service; NPR, Norwegian Patient Registry; OLS, Ordinary Least Squares; RHA, Regional Health Authority

## Additional files

Additional file 1: Table S1. Descriptive statistics for analysis variables. Changes in logged levels, by year. (DOCX 15 kb) Additional file 2: Table S2. First stage results for IV regressions. As estimated in models C in Table 2. (DOCX 16 kb) Additional file 3: Table S3. Results for interaction terms in regressions with time specific needs effects. As estimated in models E in Table 3. (DOCX 16 kb)

## Footnotes

1 [1] provides an English summary of the reform.

2 As of May 2014, the municipalities employed 5.9 % of GPs in the Medical Associations database. Thus, most GPs are contractors.

3 These are the preliminaries of a possible counterfactual argument, stating that an expansion of municipal services would have occurred *had* MCF been more comprehensive (which we, of course, do not observe). Moreover, the low cost-sharing rate (20 % of specialist service costs paid by municipalities) is usually motivated by it being an *offset* to the budgetary risks (e.g. in the face of demographic change) of the smaller municipalities (compared with the larger hospital catchment areas). Nevertheless, the argument is not very plausible. Even with a higher cost-sharing rate, the consequences, apart from heavy strains on local authorities’ revenues, could very well be a *heavier* (short-term) reliance on (readily available) specialist capabilities and a *weaker* reliance on local capabilities (i.e. costs of a “sunken” character going into “shaky” investments in “longer-term” local services). In other words, the argument may simply be that municipalities in Norway are too small for incentive schemes such as the MCF to work. We return to this point in the discussion.

4 The DRGs are DRG-462A: Complex rehabilitation, DRG-462B: Ordinary rehabilitation, DRG-462C: Other rehabilitation, DRG-462O: Unspecified rehabilitation, DRG-932O: Policlinic rehabilitation, and DRG-998O: Group-based patient recovery. The choice of DRGs is based on a presentation given by Vidar Halsteinli for the Central Norway Regional Health Authority 29.09.2010.

5 Nominal DRG prices (for the average DRG) in the 2010–2013 period are NOK 35,964 (2010), NOK 36,968 (2011), NOK 38,209 (2012), and NOK 39,447 (2013) (Norwegian Directory of Health).

6 While this is the average price per stay, as reported by the South-Eastern Norway Regional Health Authority (*Helse Sør-Øst RHF*), costs for rehabilitation stays in private institutions vary considerably, with the costliest stay priced at seven times the cheapest stay. Unfortunately, we have presently not been able to collect more precise data.

7 A standard measure for “municipal rehabilitation service” production is man-hours worked by physio- and ergotherapists and psychiatric nurses as this “is assumed to be closely related to rehabilitation”. In other words, the measure is coarser than analogues at the specialist and private level (where diagnoses, tasks, and procedures are better defined and priced). Moreover, records are not complete for a large proportion of the municipalities prior to 2013. The measure that we utilize therefore relies on the two former occupational groups (for which records are the most complete).

8 This constitutes 96 % of the 428 Norwegian municipalities. Missing values are most often associated with incomplete municipal reporting to KOSTRA.

9 Even though these results are weighted by municipal population; a point to which we return below.

10 Norwegian municipalities vary in population size from just above 200 to well over 600,000 (Oslo), and the median and mean population sizes stand at around 4500 and 12,000 respectively (2012).

11 These are the exact predictions for relative changes, i.e. exp(*α*)-1. For example, for the estimate of the *α*_2012_ effect this is exp(-0.15)-1 = 0.14. We report such exact effects in the text.

12 For example, for specialist services the estimate of α_*2012*_-α_*2011*_ = -0.15-(-0.03) = -0.12 is significant at the *p* = 0.00 level, as are the analoguos differences for the other service types.

13 Although this pattern disappears with other specifictions (model variants (B) and (C); see below).

14 This may suggest that the CR is an imprtant factor, if not strictly *the only important* determinant of behavior, in that pre- and post-reform trends are largely weak or missing altogether.

15 First stage results for two-stage least square estimations are presented in Additional file 2: Table S2.

16 For example, average (log) specialist service levels change by *α*_*2012*_-*α*_*2011*_, estimated at -0.11-(-0.02) = -0.09 from 2011 to 2012 (*z* = 2.30, *p* = 0.022). In the case of municipal rehabilitation services there are no signficant differences between years whatsoever.

17 In analyses in Table 3 we also cluster standard errors at the municipality level.

18 Although not very substantial, 2012–2013 changes, estimated by jointly testing *α*_0_ + *α*_*2013*_, are significant at the 5 % level for specialist and private services in model variant (D).

19 Implemented by adding interaction terms between the Δ*Needs* and Δ*Year* variables to equation (2). Results for interaction terms are shown in Additional file 3: Table S3.

20 For other service types (private and municipal), needs effects are alternately positive or negative, and never significant.

21 Conveniently, the additive *α* estimates may be interpreted as effects when the intreacted variable, the needs variables, are zero. In other words, *α* estimates are *Year* effects *in the absen*ce of needs changes.
