# Supplementary material for: The effects of the Norwegian Coordination Reform on the use of rehabilitation services: panel data analyses of service use, 2010 to 2013
Source: BMC Health Serv Res. 2016 Aug 5;16:353. doi: 10.1186/s12913-016-1564-6 (PMC4974745; doi:10.1186/s12913-016-1564-6)
Supplement: Additional file 1: Table S1. — Descriptive statistics for analysis variables. Changes in logged levels, by year. (DOCX 15 kb) [file 12913_2016_1564_MOESM1_ESM.docx]

**Table S1. Descriptive statistics for analysis variables (*log*[level*_t_*/level*_t-1_*]). By year (2011-2013). N=409.^†^**

| Variable | Year | Mean | St. dev. | Min. | Max. |
| --- | --- | --- | --- | --- | --- |
|  |  |  |  |  |  |
|  |  |  |  |  |  |
| Specialist level (hospital) rehabilitation services (2010 NOK per inabitant)^a^ | 2010 | -0.03 | 0.30 | -1.86 | 2.25 |
|  | 2011 | -0.12 | 0.29 | -2.32 | 1.91 |
|  | 2012 | -0.06 | 0.30 | -2.53 | 2.21 |
|  |  |  |  |  |  |
| Rehabilitation services in private institutions (2010 NOK per inhabitatnt)^b^ | 2010 | -0.07 | 0.28 | -2.31 | 2.42 |
|  | 2011 | 0.32 | 0.43 | -1.94 | 1.88 |
|  | 2012 | 0.09 | 0.38 | -2.42 | 2.17 |
|  |  |  |  |  |  |
| Municipal rehabilitation services (physiotherapist and ergotherapist man-years per 10,000 inhabitants) | 2010 | 0.07 | 0.37 | -4.28 | 3.56 |
|  | 2011 | 0.05 | 0.33 | -3.78 | 3.91 |
|  | 2012 | 0.06 | 0.29 | -2.62 | 2.89 |
|  |  |  |  |  |  |
| Share of municipal population aged over 80 | 2010 | -0.01 | 0.02 | -0.15 | 0.17 |
|  | 2011 | -0.01 | 0.02 | -0.14 | 0.19 |
|  | 2012 | -0.01 | 0.02 | -0.17 | 0.13 |
|  |  |  |  |  |  |
| Share of municipal population aged 67-79 | 2010 | 0.01 | 0.02 | -0.08 | 0.12 |
|  | 2011 | 0.03 | 0.02 | -0.09 | 0.15 |
|  | 2012 | 0.03 | 0.02 | -0.10 | 0.15 |
|  |  |  |  |  |  |
| Deaths per 1,000 inahbitants | 2010 | -0.01 | 0.15 | -1.09 | 0.81 |
|  | 2011 | 0.00 | 0.14 | -1.23 | 1.00 |
|  | 2012 | -0.03 | 0.14 | -0.96 | 0.83 |
|  |  |  |  |  |  |
| Reported crimes per 1,000 inahbitants | 2010 | -0.04 | 0.08 | -0.61 | 0.45 |
|  | 2011 | -0.02 | 0.08 | -0.66 | 0.52 |
|  | 2012 | -0.01 | 0.07 | -0.57 | 0.58 |
|  |  |  |  |  |  |
| *Source:* Norwegian Patient Registry and Statistics Norway. | |  |  |  |  |
| †) Results weighted by municipal population. | | | | | |
| a) Comprises DRG-462A: Complex rehabilitation, DRG-462B: Ordinary rehabilitation, DRG-462C: Other rehabilitation, DRG-462O: Unspecified rehabilitation, DRG-932O: Policinical rehabilitation and DRG-998O: Group based patient recovery. | | | | | |
| b) annually aggregated individual stays in privat rehabilitation institutions times average price-per-stay (NOK 2,886 in 2012). | | | | | |
