# Supplementary material for: The effects of the Norwegian Coordination Reform on the use of rehabilitation services: panel data analyses of service use, 2010 to 2013
Source: BMC Health Serv Res. 2016 Aug 5;16:353. doi: 10.1186/s12913-016-1564-6 (PMC4974745; doi:10.1186/s12913-016-1564-6)
Supplement: Additional file 2: Table S2. — First stage results for IV regressions. As estimated in models C in Table 2. (DOCX 16 kb) [file 12913_2016_1564_MOESM2_ESM.docx]

**Table S2. First stage results for IV regressions (estimated in models C in Table 2). N=1636.^†^**

|  |  | Share of pop. 67-79 (log) at *t* | |  | Share of pop.80+ (log) at *t* | |  | Deaths per inhab. (log) at *t* | |
| --- | --- | --- | --- | --- | --- | --- | --- | --- | --- |
|  |  |  |  |  |  |  |  |  |  |
|  |  |  |  |  |  |  |  |  |  |
| Y_2011_ (=1) |  | 0.015 | *** |  | -0.002 |  |  | -0.012 |  |
|  |  | (0.002) |  |  | (0.002) |  |  | (0.008) |  |
|  |  |  |  |  |  |  |  |  |  |
| Y_2012_ (=1) |  | 0.045 | *** |  | -0.007 | *** |  | -0.010 |  |
|  |  | (0.002) |  |  | (0.002) |  |  | (0.008) |  |
|  |  |  |  |  |  |  |  |  |  |
| Y_2013_ (=1) |  | 0.071 | *** |  | -0.018 | *** |  | -0.040 | *** |
|  |  | (0.003) |  |  | (0.003) |  |  | (0.008) |  |
|  |  |  |  |  |  |  |  |  |  |
| Share of pop. 67-79 (log) at *t*-3 |  | 0.382 | *** |  | 0.316 | *** |  | 0.333 | ** |
|  |  | (0.036) |  |  | (0.051) |  |  | (0.144) |  |
|  |  |  |  |  |  |  |  |  |  |
| Share of pop.80+ (log) at *t*-3 |  | 0.001 |  |  | 0.380 | *** |  | 0.271 | ** |
|  |  | (0.026) |  |  | (0.049) |  |  | (0.118) |  |
|  |  |  |  |  |  |  |  |  |  |
| Deaths per inhab. (log) at *t*-3 |  | -0.001 |  |  | -0.030 | *** |  | -0.081 | ** |
|  |  | (0.005) |  |  | (0.007) |  |  | (0.031) |  |
|  |  |  |  |  |  |  |  |  |  |
| Reported crimes per inhab. (log) |  | -0.030 | *** |  | 0.017 | * |  | -0.018 |  |
|  |  | (0.009) |  |  | (0.100) |  |  | (0.038) |  |
|  |  |  |  |  |  |  |  |  |  |
|  | | | | | | |  |  |  |
| R^2^ |  | 0.99 |  |  | 0.99 |  |  | 0.88 |  |
|  | | | | | | |  |  |  |
|  | | | | | | |  |  |  |
| †) Results weighted by municipal population. All regressions include municipality indicators (parameter estimates not shown). Reference year is 2010 (Y_2010_=1). Robust standard errors in parentheses. | | | | | | | | | |
| * p<0.10, ** p<0.05, *** <0.01. |  |  |  |  |  |  |  |  |  |
