# Supplementary material for: The effects of the Norwegian Coordination Reform on the use of rehabilitation services: panel data analyses of service use, 2010 to 2013
Source: BMC Health Serv Res. 2016 Aug 5;16:353. doi: 10.1186/s12913-016-1564-6 (PMC4974745; doi:10.1186/s12913-016-1564-6)
Supplement: Additional file 3: Table S3. — Results for interaction terms in regressions with time specific needs effects. As estimated in models E in Table 3. (DOCX 16 kb) [file 12913_2016_1564_MOESM3_ESM.docx]

**Table S3. Results for interaction terms in regressions with time specific needs effects (estimated in models E in Table 3). N=1227.^†^**

|  |  | Change from previous year in use of specialist level (hospital) rehabilitation services (log) | |  | Change from previous year in use of rehabilitation services in private institutions (log) | |  | Change from previous year in use of municipal rehabilitation services (log) | |
| --- | --- | --- | --- | --- | --- | --- | --- | --- | --- |
|  |  |  |  |  |  |  |  |  |  |
|  |  |  |  |  |  |  |  |  |  |
| Δ share of pop. 67-79 (log) × Y_2012_ (=1) |  | -1.53 |  |  | -0.92 |  |  | 0.21 |  |
|  |  | (1.24) |  |  | (1.54) |  |  | (2.02) |  |
|  |  |  |  |  |  |  |  |  |  |
| Δ share of pop.80+ (log) × Y_2012_ (=1) |  | -1.30 |  |  | -0.94 |  |  | 0.26 |  |
|  |  | (1.14) |  |  | (2.31) |  |  | (0.99) |  |
|  |  |  |  |  |  |  |  |  |  |
| Δ deaths per inhab. (log) × Y_2012_ (=1) |  | 0.11 |  |  | 0.07 |  |  | 0.27 |  |
|  |  | (0.14) |  |  | (0.21) |  |  | (0.17) |  |
|  |  |  |  |  |  |  |  |  |  |
| Δ reported crimes per inhab. (log) × Y_2012_ (=1) |  | -0.66 | ** |  | 0.05 |  |  | 0.06 |  |
|  |  | (0.29) |  |  | (0.40) |  |  | (0.33) |  |
|  |  |  |  |  |  |  |  |  |  |
| Δ share of pop. 67-79 (log) × Y_2013_ (=1) |  | -1.62 |  |  | 0.71 |  |  | -0.93 |  |
|  |  | (1.21) |  |  | (1.55) |  |  | (2.09) |  |
|  |  |  |  |  |  |  |  |  |  |
| Δ share of pop.80+ (log) × Y_2013_ (=1) |  | -1.04 |  |  | -0.96 |  |  | 1.28 |  |
|  |  | (0.98) |  |  | (1.21) |  |  | (1.10) |  |
|  |  |  |  |  |  |  |  |  |  |
| Δ deaths per inhab. (log) × Y_2013_ (=1) |  | -0.02 |  |  | 0.03 |  |  | 0.18 |  |
|  |  | (0.15) |  |  | (0.18) |  |  | (0.18) |  |
|  |  |  |  |  |  |  |  |  |  |
| Δ reported crimes per inhab. (log) × Y_2013_ (=1) |  | -0.48 |  |  | -0.18 |  |  | -0.09 |  |
|  |  | (0.32) |  |  | (0.37) |  |  | (0.33) |  |
|  |  |  |  |  |  |  |  |  |  |
|  | | | | | | |  |  |  |
| R^2^ |  | 0.03 |  |  | 0.16 |  |  | 0.01 |  |
|  | | | | | | |  |  |  |
|  | | | | | | |  |  |  |
| †) Results weighted by municipal population. Robust standard errors clustered at the municipality level in parentheses. | | | | | | | | | |
| * p<0.10, ** p<0.05, *** p<0.01. |  |  |  |  |  |  |  |  |  |
